# Models to predict the surgical outcome of mini-ECIRS (endoscopic combined intrarenal surgery) for renal and/or ureteral stones

**DOI:** 10.1038/s41598-023-50022-6

**Published:** 2023-12-21

**Authors:** Hiroki Ito, Kentaro Sakamaki, Tetsuo Fukuda, Fukashi Yamamichi, Takahiko Watanabe, Tadashi Tabei, Takaaki Inoue, Junichi Matsuzaki, Kazuki Kobayashi

**Affiliations:** 1 Department of Urology, Yokosuka Kyosai Hospital, Yokosuka, Japan; 2 Department of Urology, Yokohama City University Graduate School of Medicine, Yokohama, Japan; 3 Faculty of Health Data Science, Juntendo University, Tokyo, Japan; 4 Department of Urology, Ohguchi East General Hospital, Yokohama, Japan; 5 Department of Urology, Hara Genitourinary Hospital, Kobe, Japan

**Keywords:** Medical research, Risk factors, Urology

## Abstract

To establish a safer and more efficient treatment strategy with mini-endoscopic combined intrarenal surgery (ECIRS), the present study aimed to develop models to predict the outcomes of mini-ECIRS in patients with renal and/or ureteral stones. We retrospectively analysed consecutive patients with renal and/or ureteral stones who underwent mini-ECIRS at three Japanese tertiary institutions. Final treatment outcome was evaluated by CT imaging at 1 month postoperatively and stone free (SF) was defined as completely no residual stone or residual stone fragments ≤ 2 mm. Three prognostic models (multiple logistic regression, classification tree analysis, and machine learning-based random forest) were developed to predict surgical outcomes using preoperative clinical factors. Clinical data from 1432 ECIRS were pooled from a database registered at three institutions, and 996 single sessions of mini-ECIRS were analysed in this study. The overall SF rate was 62.3%. The multiple logistic regression model consisted of stone burden (*P* < 0.001), number of involved calyces (*P* < 0.001), nephrostomy prior to mini-ECIRS (*P* = 0.091), and ECOG-PS (*P* = 0.110), wherein the area under the curve (AUC) was 70.7%. The classification tree analysis consisted of the number of involved calyces with an AUC of 61.7%. The random forest model showed that the top predictive variable was the number of calyces involved, with an AUC of 91.9%. Internal validation revealed that the AUCs for the multiple logistic regression model, classification tree analysis and random forest models were 70.4, 69.6 and 85.9%, respectively. The number of involved calyces, and a smaller stone burden implied a SF outcome. The machine learning-based model showed remarkably high accuracy and may be a promising tool for physicians and patients to obtain proper consent, avoid inefficient surgery, and decide preoperatively on the most efficient treatment strategies, including staged mini-ECIRS.

## Introduction

Endoscopic combined intrarenal surgery (ECIRS) is a combination of endoscopic surgery with percutaneous nephroscopic access and retrograde ureteroscopic access, and is now recognised as one of the standard endoscopic treatment options for renal and ureteral stone^1–4^. Achieving stone free (SF) status rates are 65.3–81.9%^2,5,6^ and perioperative complication rates are 7.3–38.6%^2,7,8^ indicating higher efficacy and a safer procedure than percutaneous nephrolithotripsy (PCNL) alone^1,4,9–11^. In the last decade, ECIRS has become a less invasive procedure, and mini-ECIRS has emerged with a smaller percutaneous access tract and nephroscopy, demonstrating the advantages of less postoperative pain and potentially less bleeding than conventional ECIRS^12^.

However, ECIRS requires at least two experienced endoscopic surgeons and is generally challenging to perform safely because of the learning curve^13^, which is reported to cause perioperative complications, including fever, sepsis, and other organ injuries^2,7,8^. There is a clear demand for accurate prediction of ECIRS outcomes based on preoperative factors, allowing the allocation of reasonable and feasible treatment strategies. However, there is scarce literature reporting on and investigating mini-ECIRS, leading to a lack of clinical evidence for ECIRS.

To address some clinical issues in ECIRS, we created the largest cohort of ECIRS data from three high-volume centres in Japan and developed three prediction models of mini-ECIRS outcomes based on clinical parameters, followed by internal validation. To the best of our knowledge, this is the first study to develop prediction models of mini-ECIRS outcome including machine learning model utilizing the largest cohort of the database. Those our proposed prediction model will lead to a safer and more efficient treatment strategy with mini-ECIRS and will be beneficial for surgeons and patients who undergo mini-ECIRS.

## Materials and methods

The clinical information of consecutive patients who underwent conventional or mini-ECIRS for urinary stone disease between 2015 and 2021 at three high-volume centres in Japan (Yokosuka Kyosai Hospital, Ohguchi East General Hospital, and Hara Genitourinary Hospital) was collected and pooled for analysis. The inclusion criterion was single-session mini-ECIRS for the treatment of renal and/or ureteral stones. There were no restrictions on patient age, Eastern Cooperative Oncology Group Performance Status (ECOG-PS), and position setup during mini-ECIRS. Both modified Valdivia and prone positions were included, as previous work has shown ECIRS in both positions with comparable results^14^. The exclusion criteria were as follows: preoperatively intended staged procedures, no available postoperative CT, and no available preoperative clinical information. Patients with urinary tract abnormalities, including horseshoe kidney, ileal conduit, neobladder, severe ureteral stricture, and ureteropelvic junction (UPJ) obstruction that did not allow ureteroscopy (URS) to be used during mini-ECIRS were also excluded. The study protocol (IRB number 20-90 at Yokosuka Kyosai Hospital, 202201 at Ohguchi East General Hospital, and 2021-05-06 at Hara Genitourinary Hospital) waiving the requirement for written informed consent was approved by the institutional ethics committee of each hospital. Informed consent was obtained from participants as an opt-out on each hospital website. The study was conducted in accordance with the principles of the Declaration of Helsinki and all local regulations.

Non-contrast CT imaging was utilised to evaluate surgical outcomes at 1 month postoperatively, following the same protocol in the three institutions. SF was defined by CT imaging 1 month postoperatively as completely no residual stone or residual stone fragments ≤ 2 mm. Residual stone fragments > 2 mm was defined as non-SF.

The ECIRS technique was similar to previously reported methods in modified Valdivia or prone position^15^. All enrolled procedures were performed by 2–3 urologists, with at least one surgeon with more than 50 ECIRS procedures as a supervisor. In brief, two urologists worked simultaneously to fragment the kidney stones: one performed PCNL and the other performed URS. A ureteral access sheath (10/12 or 12/14Fr B-Flex, Rocamed, Monaco, Italy) was placed to facilitate frequent insertion of fURS (URF-V2 or P-6™; Olympus, Tokyo, Japan or Flex-X2™, Karl Storz, Tuttlingen, Germany). A 9.5Fr ureteral access sheath (Flexor, Cook Medical LLC, Bloomington, USA) was used for difficult ureters^16^. Percutaneous renal access was performed under ultrasound guidance with or without fURS monitoring. The choice of puncture calyx was at the discretion of the PCNL surgeon for efficient treatment. The percutaneous operating sheath (13Fr or 17.5F) was inserted using a one-step dilator. We utilized a Holmium‐YAG laser (Lumenis Pulse™ 120H; Yokne’am Illit, Israel or Dornier MedTech; Munich, Germany) through a fURS (commonly used laser settings were 0.5–1.0 J at 5–10 Hz) and a pneumatic lithotripsy (Swiss LithoClast® Master J; Electro Medical Systems, Nyon, Switzerland) through a mini-nephroscope (Olympus or Karl Storz). The stone burdens were crashed into tiny fragments and washed out through the PCNL sheath using manual or automated retrograde irrigation (UROMAT E.A.S.I.®; Karl Storz). Finally, a 6F ureteral stent tube and/or a 14F urinary nephrostomy tube were placed for 2–4 weeks and/or a few days, respectively, in cases of risk of postoperative infection or bleeding.

Three prognostic models (multiple logistic regression, classification tree analysis, and machine learning-based random forest) were used to predict SF using preoperative clinical factors^17^. The models were developed using age, sex, body mass index (BMI), ECOG-PS, stone laterality (left or right), number of stones, stone burden (sum of the largest stone diameters), number of involved calyces, stone position (presence or absence of R2 stone), presence of hydronephrosis, indwelling preoperative stenting, and preoperative nephrostomy. To summarise the crude relationship, a univariate logistic regression was initially performed. In multiple logistic regression, variables were selected by stepwise selection using the Akaike Information Criteria^18^. The area under the receiver operating characteristic curve (AUC) was used to evaluate the predictive ability. The random forest model is a supervised machine learning algorithm that grows multiple classification trees, wherein variables are evaluated for more accurate prediction using the mean decrease in the Gini coefficient value (a larger mean decrease indicates stronger predictive potential)^17,19,20^. Classification tree analysis enables the selection of predominant factors and determines the significance thresholds of these parameters^17,21^. Internal validation was performed to evaluate overfitting and optimism using the bootstrap method^22^, wherein 1000 bootstrap samples were repeatedly generated from an original dataset. All analyses were performed using R 4.2.3, using the rpart, randomForest, and pROC packages.

### Informed consent

Informed consent was obtained from participants as an opt-out on each hospital website. The study protocol (IRB number 20-90 at Yokosuka Kyosai Hospital, 202201 at Ohguchi East General Hospital, and 2021-05-06 at Hara Genitourinary Hospital) waiving the requirement for written informed consent was approved by the institutional ethics committee of each hospital.

## Results

**Figure 1:**
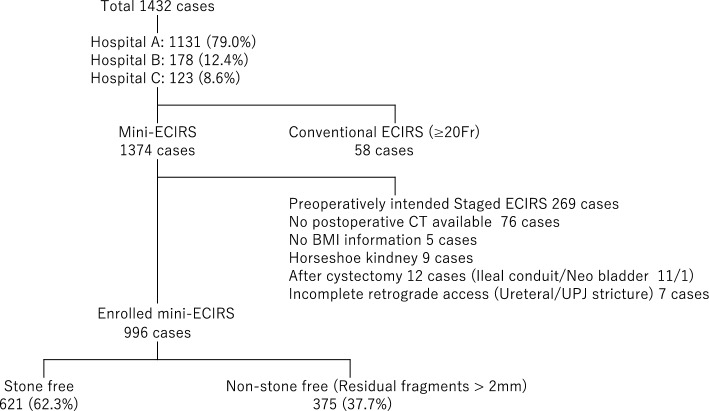
Flow chart of selection.

Clinical data from 1432 ECIRS were pooled in a database registered from three institutions and 1374 of 1432 were identified as mini-ECIRSs. Finally, 996 single sessions of mini-ECIRS were analysed in this study after 378 cases were excluded, mainly due to intended preoperative staged ECIRS (Fig. 1). The overall SF rate (complete no residual stone or residual stone fragments ≤ 2 mm) was 62.3%. Only three cases were performed in the prone position and remained in the modified Valdivia position. The stone burden, number of stones, and involved calyces were significantly lower in the SF group than in the non-SF group (*P* < 0.001, Table 1).

**Table 1:** Comparison of patient characteristics between the stone free and non-stone free groups of the mini-ECIRS.

Parameter		SF group	Non-SF group	*P* value
Number of patients		621	375	
Age		56.9 ± 12.8	57.5 ± 13.2	0.514
Sex	Female	191 (30.8%)	117 (31.2%)	0.883
	Male	430 (69.2%)	258 (68.8%)	
BMI		24.2 ± 4.6	24.2 ± 4.7	0.464
ECOG-PS	0	587 (94.5%)	354 (94.4%)	0.395
	1	17 (2.7%)	15 (4.0%)	
	2	7 (1.1%)	1 (0.3%)	
	3	2 (0.3%)	2 (0.5%)	
	4	8 (1.3%)	3 (0.8%)	
Stone laterality	Left	322 (51.9%)	218 (58.1%)	0.054
	Right	299 (48.1%)	157 (41.9%)	
Stone burden (mm)		29.1 ± 13.8	39.6 ± 24.7	< 0.001
Number of stone	Average	2.5 ± 2.0	3.3 ± 2.5	< 0.001
	Median	2 (1–12)	2 (1–20)	< 0.001
Number of involved calyces	Average	1.5 ± 1.3	2.7 ± 1.8	< 0.001
	Median	1 (0–8)	2 (0–8)	< 0.001
Stone location	Including R2 stone	488 (78.6%)	335 (89.3%)	< 0.001
	Only R3 and/or ureteral stone	133 (21.4%)	40 (10.7%)	
Presence of hydronephrosis		417 (67.1%)	231 (61.6%)	0.075
Presence of prestenting		185 (29.8%)	118 (31.5%)	0.578
Presence of nephrostomy		44 (7.1%)	34 (9.1%)	0.259
Presence of microhematuria		427 (68.8%)	262 (69.9%)	0.981
History of urinary tract infection		92 (14.8%)	53 (14.1%)	0.768

ECIRS, endoscopic combined intrarenal surgery; ECOG-PS, Eastern Cooperative Oncology Group Performance Status; SF, stone free.

Surgical treatment outcomes are summarised in Table 2. The most common postoperative complications (Clavien–Dindo ≥ 2) were fever, sepsis, renal vascular complications, and other organ injuries. Fever (*P* = 0.0021) and sepsis (*P* = 0.001) were more frequent in the non-SF cases than in the SF cases.

**Table 2:** Comparison of perioperative surgical outcomes between the stone free and non-stone free groups of the mini-ECIRS.

Parameter		SF group (N = 621)	Non-SF group (N = 375)	*P* value
Operation time (min)		104.9 ± 34.6	120.0 ± 30.6	< 0.001
Number of tracts	1	620	362	< 0.001
	2	0	11	
	3	1	1	
Indwelling postoperative stenting		375 (60.4%)	267 (71.2%)	< 0.001
Placement of postoperative nephrostomy		423 (68.1%)	293 (78.1%)	< 0.001
Duration of hospital stay (days)		5.2 ± 2.2	5.9 ± 3.6	< 0.001
Serum Hemoglobin	Preoperative	14.3 ± 1.7	14.1 ± 1.7	0.142
	One day postoperative decline	− 1.3 ± 0.9	− 1.4 ± 1.1	0.162
Stone composition analysis	Calcium oxalate	246 (39.6%)	157 (41.9%)	0.006
	Calcium phosphate	40 (6.4%)	20 (5.3%)	
	Uric acid	15 (2.4%)	6 (1.6%)	
	Magnesium ammonium phosphate	6 (1.0%)	6 (1.6%)	
	Mixed	288 (46.4%)	169 (45.1%)	
	Unclear	26 (4.2%)	17 (4.5%)	
Surgical complications	Fever	151 (24.3%)	116 (30.9%)	0.0021
	Sepsis	5 (0.8%)	14 (3.7%)	0.001
	Renal vascular complication	4 (0.6%)	1 (0.3%)	0.723
	Other organ injury	4 (0.6%)	1 (0.3%)	0.723
	Bleeding neccesiating blood transfusion	1 (0.2%)	3 (0.8%)	0.128

ECIRS, endoscopic combined intrarenal surgery; SF, stone free.

**Figure 2:**
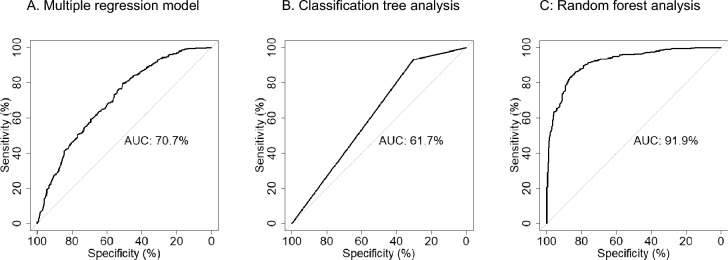
Receiver operating characteristics (ROC) curve of 3 models predicting stone free outcome of mini-ECIRS (residual fragments ≤ 2 mm).

Univariate logistic regression analysis revealed that the significant predictive variables were the number of stones, stone burden, number of calyces involved, and stone position (*P* < 0.001 in all four, Table 3). The multiple logistic regression model consisted of stone burden (*P* < 0.001), number of involved calyces (*P* < 0.001), presence of nephrostomy prior to mini-ECIRS (*P* = 0.091), and ECOG-PS (*P* = 0.110). The AUC was 70.7% (Table 3 and Fig. 2A).

**Table 3:** Univariate and multivariate logistic regression models for predicting stone free after mini-ECIRS.

Parameter		Univariate analysis					Multivariate analysis				
		Odds ratio	Lower CI	Upper CI	*P* value	AUC	Odds ratio	Lower CI	Upper CI	*P* value	AUC
Age		0.997	0.987	1.007	0.520	0.512					0.707
Gender		1.021	0.773	1.346	0.883	0.502					
BMI		1.001	0.974	1.029	0.927	0.492					
ECOG-PS	1–4 versus 0	0.976	0.562	1.733	0.933	0.501	1.703	0.897	3.333	0.11	
Stone laterality	Left versus right	1.289	0.996	1.671	0.054	0.531					
Number of stones		0.861	0.810	0.913	< 0.001	0.591					
Stone burden		0.970	0.962	0.977	< 0.001	0.634	0.983	0.975	0.992	< 0.001	
Number of involved calyces		0.606	0.549	0.665	< 0.001	0.695	0.653	0.588	0.723	< 0.001	
Stone position	With versus without R2	0.438	0.297	0.635	< 0.001	0.554					
Presence of hydronephrosis		1.274	0.975	1.664	0.075	0.528					
Presence of prestenting		0.924	0.701	1.221	0.578	0.508					
Presence of nephrostomy		0.765	0.480	1.227	0.261	0.510	0.634	0.374	1.082	0.091	

ECIRS, endoscopic combined intrarenal surgery; CI, confidence interval; AUC, area under the receiver operating characteristic curve; BMI, body mass index; ECOG-PS, Eastern Cooperative Oncology Group Performance Status.

**Figure 3:**
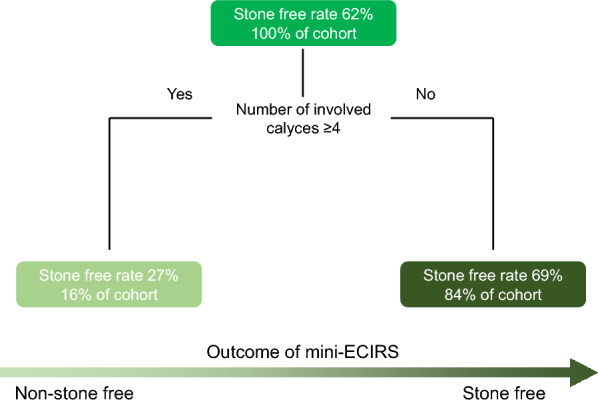
Classification tree analysis to predict mini-ECIRS outcome.

The classification tree analysis consisted of the number of calyces involved, with an AUC of 61.7% (Fig. 2B). The threshold value for the number of involved calyces was four (Fig. 3). The random forest model showed that the top predictive variable was the number of involved calyces, followed by BMI, stone burden, age, and number of stones (Table 4). The model involving all clinical variables showed an AUC of 91.9% (Fig. 2C). Internal validation using the bootstrap method showed that the AUCs of the logistic regression model, classification tree analysis, and random forest model were 70.4% (95% confidential interval (CI) [0.695–0.711]), 69.6% (95% CI [0.612–0.743]), and 85.9% (95% CI [0.840–0.876]), respectively (Table 5).

**Table 4:** Random forest model for predicting stone free after mini-ECIRS.

Parameter	Mean decrease in the Gini coefficient	AUC
Age	27.633	0.919
Gender	3.346	
BMI	33.371	
ECOG-PS	2.142	
Stone laterality	3.737	
Number of stones	13.137	
Stone burden	32.269	
Number of involved calyces	41.374	
Stone position	6.585	
Presence of hydronephrosis	2.910	
Presence of prestenting	3.667	
Presence of nephrostomy	1.980	

ECIRS, endoscopic combined intrarenal surgery; AUC, area under the receiver operating characteristic curve; BMI, body mass index; ECOG-PS, Eastern Cooperative Oncology Group Performance Status.

**Table 5:** AUC of internal validation by bootstrap method of 3 models.

	Real data	Bootstrap sample		
		Average	2.5% point	97.5% point
Multilogistic regression model	0.707	0.704	0.695	0.711
Classification tree analysis	0.617	0.696	0.612	0.743
Random Forest	0.919	0.859	0.840	0.876

AUC, area under the receiver operating characteristic curve.

## Discussion

The current study developed three different statistical models to predict SF in mini-ECIRS. The multiple logistic regression model consisted of four clinical parameters: stone burden, number of involved calyces, and presence of nephrostomy before mini-ECIRS, and ECOG-PS. Classification tree analysis showed that the number of calyces involved, in particular when less than four, was significantly associated with SF. Random forest analysis, a machine learning-based model, showed remarkably high accuracy of outcome prediction based on preoperative parameters in both real and internal validation datasets. To our knowledge, this multicentre cohort is the largest in the mini-ECIRS database.

A key finding of the study was that all three prediction models consistently demonstrated that the number of involved calyces was the most important predictor of mini-ECIRS outcomes. In particular, if the number of involved calyces is < 4, the outcome seems to be favourable, and a single session of mini-ECIRS should be recommended as first-line treatment. Otherwise, multiple staged sessions of the mini-ECIRS might be better. Enrolled mini-ECIRSs were mostly single tract surgery and multi-tract ECIRS might be reasonable options in case the number of involved calyces was ≥ 4. The number of calyces involved has been reported to be an independent predictor of PCNL^23^ and conventional ECIRS outcome^6^. Another study showed that the number of calyces involved was an independent predictor of postoperative complications in ECIRS^8^. Interestingly, those papers consistently indicated that a clinically significant cut-off causing worse outcomes were ≥ 4 involved calyces. These findings might suggest that ≥ 4 involved calyces is the limit for retrograde assistant during ECIRS, such as reposition and dusting with URS.

In addition to the number of calyces involved, stone burden was a strong predictor of mini-ECIRS in this study. Stone burden was identified as a long-term treatment outcome predictor in stone surgeries, including ECIRS^24^, PCNL^23,25^ and retrograde URS^26,27^. However, the current study showed that stone burden was the predominant factor in the prediction model of mini-ECIRS outcomes.

Before this investigation, we considered the presence of hydronephrosis as a possible successful predictor, due to hydronephrosis allowing us easier, safer, and more appropriate percutaneous access. However, the three statistical models currently used did not identify hydronephrosis as a successful indicator. These findings may suggest that easier development of access did not always indicate complete stone removal, wherein hydronephrosis was possibly a confounder of other variables, or the current analysis of mixed renal and ureteral stones may have caused heterogeneity in this cohort, resulting in hydronephrosis having less impact. Another interesting finding was that multivariate analysis showed that BMI was not a significant predictor of mini-ECIRS outcomes; however, random forest analysis indicated that BMI was one of the best predictors as well as number of involved calyces and stone burden. The possible reason for these discrepancies in BMI was the statistical character of the random forest analysis, which counts continuous values as more significant than categorical values^19–21^. Indeed, no previous studies have shown that high BMI contributes to worse outcomes of PCNL or ECIRS, despite some speculation that high BMI results in difficulties in setting patient positioning during surgery and decreased visibility of the pelvis when puncturing.

In other clinical areas, machine learning and deep learning techniques show outstanding performance^28^, with recent machine learning models demonstrating high accuracy in disease detection and prediction of surgical outcomes^29,30^. Our random forest model, based on machine learning techniques, showed remarkably high accuracy of more than 90% for real data and more than 85% for internal validation, which is better than the other two models, suggesting that the model is effective.

The limitations of this study are its retrospective data analysis and the inclusion of surgeries performed by multiple surgeons. However, we were of the opinion that this study was a good reflection of the real-world practice. CT value could not be assessed in this study because of different CT imaging facilities among multi-centre. External validation of prediction models is warranted to determine whether the current model is consistent and reliable.

In conclusions, the three models predicted the successful outcomes of single session mini-ECIRS using preoperative parameters from the largest mini-ECIRS database. Less than four involved calyces and a smaller stone burden implied a successful outcome. The machine learning-based model showed remarkably high accuracy and may be a promising tool for physicians and patients to obtain proper consent, avoid inefficient surgery, and decide preoperatively on the most efficient treatment strategies, including staged mini-ECIRS.

### Supplementary Information

Supplementary Information.

## Data Availability

Statistical codes were available and attached as a Supplementary data to this article. The datasets generated and analyzed during the current study is not publicly available but are available from the corresponding author on reasonable request.
